# The Peripheral Inflammatory Response to Alpha-Synuclein and Endotoxin in Parkinson's Disease

**DOI:** 10.3389/fneur.2018.00946

**Published:** 2018-11-20

**Authors:** Alice J. White, Ruwani S. Wijeyekoon, Kirsten M. Scott, Nushan P. Gunawardana, Shaista Hayat, I. H. Solim, H. T. McMahon, Roger A. Barker, Caroline H. Williams-Gray

**Affiliations:** John van Geest Center for Brain Repair, Department of Clinical Neurosciences, University of Cambridge, Cambridge, United Kingdom

**Keywords:** Parkinson's disease, immune system, alpha-synuclein, endotoxin, cytokines

## Abstract

The immune system is activated in Parkinson's Disease (PD), as evidenced by neuroinflammatory changes within the brain as well as elevated immune markers in peripheral blood. Furthermore, inflammatory cytokine levels in the blood are associated with disease severity and rate of progression. However, the factors driving this immune response in PD are not well established. We investigated cell-extrinsic factors in systemic immune activation by using α-synuclein monomers and fibrils, as well as bacterial toxins, to stimulate peripheral blood mononuclear cells (PBMCs) derived from 31 patients and age/gender-matched controls. α-synuclein monomers or fibrils resulted in a robust cytokine response (as measured by supernatant cytokine concentrations and mRNA expression in cultured cells) in both PD and control PBMCs, similar to that induced by bacterial LPS. We found no PD vs. control differences in cytokine production, nor in mRNA expression. Levels of endotoxin within the recombinant α-synuclein used in these experiments were very low (0.2–1.3EU/mL), but nonetheless we found that comparable levels were sufficient to potentially confound our cytokine concentration measurements for a number of cytokines. However, α-synuclein monomers increased production of IL-1β and IL-18 to levels significantly in excess of those induced by low-level endotoxin. In conclusion, this study: (i) highlights the importance of accounting for low-level endotoxin in antigen-PBMC stimulation experiments; (ii) indicates that cell-extrinsic factors may be a major contributor to immune activation in PD; and (iii) suggests that α-synuclein may play a role in inflammasome-related cytokine production in the periphery.

## Introduction

The immune system is known to be altered in Parkinson's disease (PD). Whilst some of these changes may be secondary phenomena, a growing body of evidence suggests that the immune system may play a contributory role in the primary progression of PD (1, 2). α-synuclein is the key protein implicated in the pathogenesis of PD, forming intracellular aggregates known as Lewy bodies (3). Fibrillar α-synuclein is the principal pathological form present in Lewy bodies (4), but the protein also exists in monomeric and oligomeric forms within the CNS, and all three may trigger a central immune response orchestrated by microglia (5–7). Mutations in α-synuclein are known to be associated with PD risk (8) and *in-vitro* studies of the behavior of monocytes and microglia stimulated with mutant α-synuclein monomers demonstrate increased cytotoxic immune responses in comparison to wild-type α-synuclein-exposed cells (9, 10). Components of α-synuclein have also been shown to lead to activation of T-lymphocytes and monocytes (11, 12), all of which suggests that α-synuclein may drive both a neuronal pathology and an inflammatory process in PD. Overproduction of cytokines in PD perpetuates the inflammatory response centrally and systemically (13). Serum cytokines [for example, IL-1ß, IL-2, IL-10, IFNγ, and TNF-α (14, 15)] and peripheral blood mononuclear cell (PBMC) cytokine production has been correlated with PD symptom severity (16, 17) and rate of disease progression (2). The question therefore arises: could this be driven by α-synuclein in the periphery?

Aberrant α-synuclein is distributed throughout peripheral organs, blood, interstitial and extracellular fluids in PD (18–20) and may act as a catalyst for activation of the peripheral immune system (1). Indeed, selected α-synuclein peptides stimulate a specific T-cell response in 40% of patients, via presentation by MHC alleles which are known to be genetically associated with PD risk (11). Fibrillar α-synuclein has been shown to act via Toll-like receptor (TLR) and inflammasome pathways in monocytes leading to IL-1β production (12).

However, other factors such as infections or translocation of bacterial toxins from the gut may also contribute to inflammation in PD (21, 22). For example, lipopolysaccharides (LPS) stimulate PBMCs via the TLR and inflammasome pathways to produce an inflammatory cytokine response (12), and several studies have investigated this response in PD albeit with inconsistent results. LPS-stimulated cytokine production has been reported to be elevated in PD compared to controls, along with the basal production of some cytokines (IL-1ß, IFNγ, and TNF-α) (16). A second study showed that production of IL-1ß, IL-6, and TNF-α is enhanced in PD PBMCs, while IL-2 is reduced (23). However, in another study, production of IFNγ by LPS-stimulated PBMCs was lower in patients than controls, while IL-6, IL-1α, and IL-1ß levels were no different, but decreasing concentrations correlated worsened disease severity (24).

Given this ambiguity in the literature and the absence of any study investigating both α-synuclein and LPS stimulation of PBMCs in PD patients, we sought to understand how stimulation by α-synuclein monomers, fibrils, and LPS affects PBMC cytokine production in PD patients and matched controls.

## Methods

Early-stage PD patients (Hoehn and Yahr ≤2), fulfilling UK PD Brain Bank Criteria, aged 55–80, were recruited from the PD Research Clinic at the John van Geest Center for Brain Repair, Cambridge. A movement disorder accredited neurologist conducted clinical and neuropsychological assessments.

Age and gender matched control participants were recruited from the NIHR Cambridge Bioresource (http://www.cambridgebioresource.org.uk) and had no history of neurological disease, self-reported memory problems, or depression. Ethical approval was obtained from the East of England-Cambridge Central Research Ethics Committee (REC 03/303). Exclusion criteria were: other neurodegenerative disorders, chronic inflammatory or autoimmune disorders, current clinically significant infection or use of anti-inflammatory/immunomodulatory medications, surgery within the last month, or recent vaccinations. Data from this cohort also contributed to our previously published study (25).

PBMCs were extracted from venous blood by centrifugation over a Ficoll gradient, washed and cultured (37°C, 5% CO_2_) for 24 h in RPMI (Life Technologies) and 10% fetal calf serum (FCS, Sigma) in aliquots of 1 million cells per mL per well, either unstimulated, or with LPS (1 ng/mL), α-synuclein monomers (2 nmol/mL) or fibrils (2 nmol/mL). Supernatant was collected and stored at −80°C, and cultured PBMCs were washed and stored in RNA protect (Qiagen) at −80°C. Matched samples were processed in parallel.

Recombinant α-synuclein was produced by expression in *E.coli* Rosetta using human α-synuclein cDNA, and aggregates were confirmed on SDS-PAGE gel (Supplementary Methods and Supplementary Figure 1). Endotoxin levels were determined using LAL assays (Lonza Verviers SPRL, Belgium).

Cytokines (IFNγ, IL-1β, IL-2, IL-4, IL-6, IL-8, IL-10, IL-12p70, IL-13, TNF-α) were measured in culture supernatants using the Meso Scale Discovery (Rockville) platform V-Plex Pro-inflammatory panel 1 electrochemiluminescence assay. Secondary analyses were performed in a subset of samples/conditions to measure IL-18 (MSD U-PLEX Human IL-18 assay) and caspase-1 (Human caspase-1/ICE Quantikine ELISA kit, R&D Systems) as markers of inflammasome activation. Assays were run according to manufacturer's instructions. Supernatant samples were diluted 1:10 or 2:3 in the appropriate buffer and assayed in duplicate. Cytotoxicity post-culture was quantified with the Pierce LDH Cytotoxicity Assay Kit (ThermoFisher).

RNA was extracted from cultured PBMCs using the RNeasy Mini Kit (Qiagen) according to the manufacturer's instructions. RNA was reverse-transcribed using SuperScript™ III First-Strand Synthesis SuperMix for qRT-PCR (ThermoFisher Scientific). TaqMan Real-Time PCR was used for quantification of gene expression, and primers were IL-6 (HS00174131M1) and IL-1ß (HS001555410M1) (ThermoFisher Scientific). Assays were run in triplicate. Relative quantification was carried out on a QuantStudio 12K Flex Real-Time PCR machine and calculated using comparative cycle threshold (ΔΔCT method) relative to the housekeeping gene GAPDH, and a randomly selected endogenous control common to all plates.

Cytokine concentrations across antigens and PD status were compared using repeated-measures ANOVAs, and mRNA production using 2-way ANOVAs (GraphPad Prism version 7, SPSS version 25). Outliers were removed using Grubbs' tests (*p* < 0.05).

## Results

31 PD patients [mean disease duration 4.3 (1.1) years] and 31 controls (matched for age and gender) were included (Supplementary Table 1).

PBMCs were cultured with LPS (*n* = 31 case-control pairs), and α-synuclein monomers and fibrils (*n* = 19 case-control pairs). A subset of α-synuclein-cultured PBMCs were used for the gene expression assays based on RNA availability.

**Figure 1:**
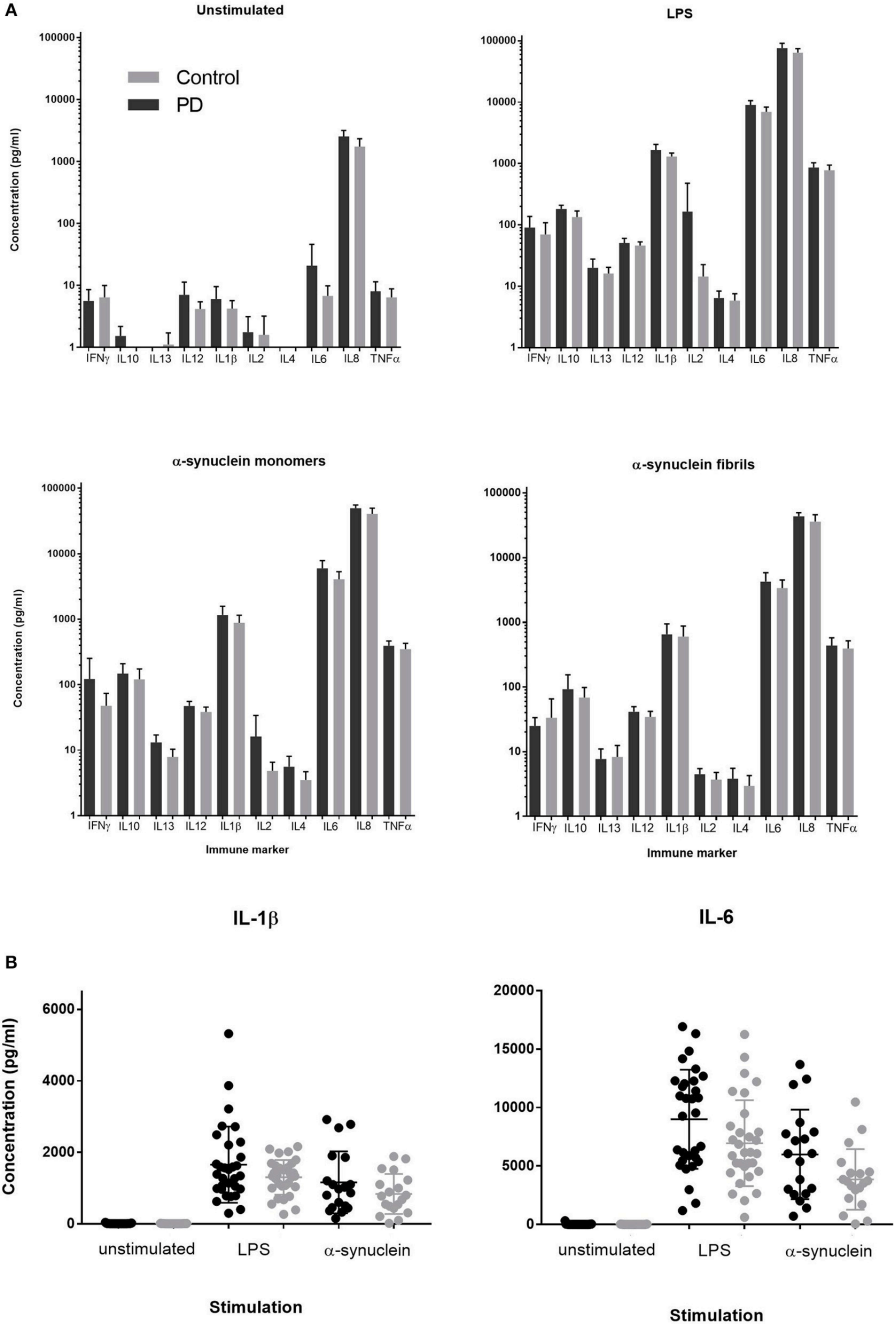
**(A)** Supernatant cytokine concentration produced by PD and control PBMCs cultured for 24 h in media containing LPS (1ng/ml), α-synuclein fibrils, or α-synuclein monomers (1 nmol = 27.3 ng/ml). **(B)** Individual level data for IL-1β and IL-6 concentrations in post-culture supernatant in PD cases and matched controls for direct comparison between unstimulated, LPS and α-synuclein monomer stimulation for two key inflammatory cytokines. Error bars represent SEM.

Stimulation with either LPS, α-synuclein monomers or fibrils led to robust cytokine stimulation compared to the unstimulated condition (*p* < 0.0001, RMANOVA, main effect of antigen). There was no main effect of patient vs. control status on cytokine production for any antigen (*p* > 0.05, RMANOVA; Figure 1). PBMC supernatant LDH levels were not different between α-synuclein, LPS, or unstimulated cultures. Expression of IL-6 and IL-1β was quantified by qRT-PCR, given that these cytokines showed the greatest PD-control differences on LPS stimulation in previous studies (16, 24). IL-6 and IL-1β expression were elevated in response to stimulation by both LPS and α-synuclein (2-way ANOVA and *post-hoc* Tukey's test *p* < 0.0001; Gene expression, relative units mean ± standard deviation(SD), IL-6: unstimulated PD = 2.18 ± 4.28, Control = 1.67 ± 2.82; α-synuclein PD = 929.72 ± 485.93, Control = 845.05 ± 419.67; LPS PD = 989.05 ± 968.66, Control = 810.00 ± 731.10, IL-1β: unstimulated PD = 2.99 ± 3.72, Control = 2.54 ± 3.76; α-synuclein PD = 207.01 ± 95.13, Control = 171.39 ± 60.10; LPS PD = 155.59 ± 116.50, Control = 136.77 ± 86.64). There was no main effect of disease status between PD and control groups (*p* > 0.05, two-way ANOVA).

**Figure 2:**
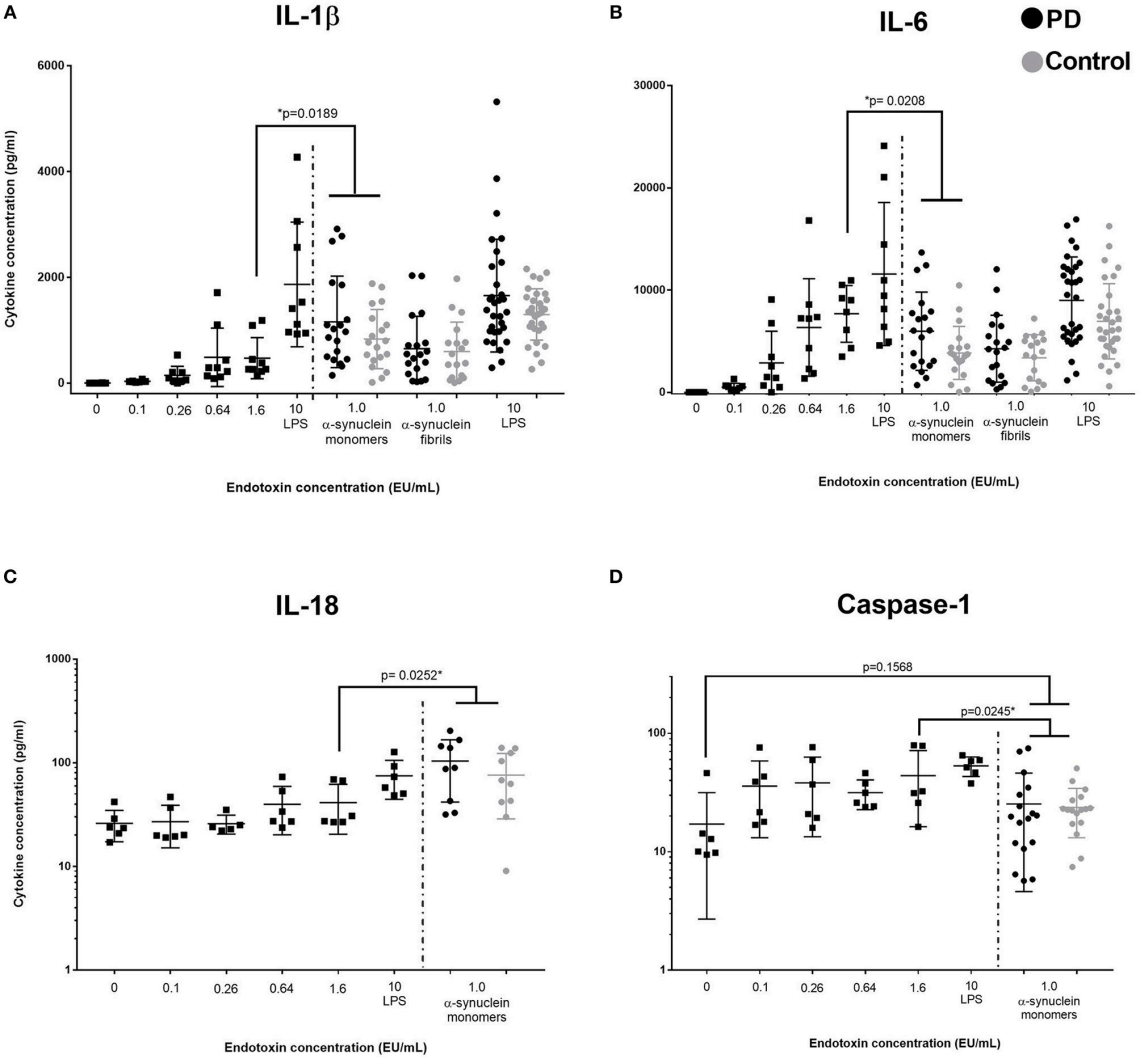
Comparison of low concentration endotoxin and α-synuclein stimulation of PBMCs. Squares represent cytokine concentrations from PBMC cultures with varying concentrations of endotoxin (**A, B**: *n* = 9, 5 PD and 4 controls, **C,D**: *n* = 6, 3PD and 3 controls, assayed in duplicate; left of dashed line). Circles represents PD (black) and control (gray) concentrations in post-culture supernatants from the original assays cultured with α-synuclein monomers, fibrils, or LPS (α-synuclein conc: 1 nmol = 27.3 ng/ml, LPS conc: 1 ng/ml). *P*-values indicates comparison between 1.6 EU/ml (comparable to endoxin level in our α-synuclein preparation) and grouped PD/control cohort stimulated by α-synuclein monomers (No PD/control differences were observed in post-culture cytokine concentrations) Bars represent mean value and first and third quartiles. **(A)** Data suggest that IL-1β production is stimulated by α-synuclein in excess of stimulation by the equivalent value of endotoxin present as a contamiant. **(B)** IL-6 (and all other measured cytokines) do not show this increased production. Data indicates that endotoxin is the primary driver of elevated IL-6 concentration, rather tha α-synuclein. **(C)** IL-18 prodution is also significantly increased in response to α-synuclein stimulation, compared to stimulation with an endotoxin concentration comparable to contaminating levels. **(D)** Caspase-1 levels are not significantly increased by α-synuclein stimulation compared to the unstimulated condition.

Given that α-synuclein produced a similar magnitude of cytokine response to LPS, we examined the α-synuclein for the presence of any associated endotoxin. Despite procedures to remove contaminating endotoxin as detailed in the methods, endotoxin concentrations in samples at 2 nmol/mL were 0.2–1.3EU/mL on testing multiple aliquots (Lonza). LPS (1 ng/mL) contained >10 EU/mL. To ascertain whether the levels of contaminating endotoxin were sufficient to confound cytokine measurements in the α-synuclein cultures, we used six endotoxin standard dilutions (0, 0.1, 0.26, 0.64, 1.6, and LPS>10EU/mL) to stimulate PBMCs using otherwise identical conditions (PD *n* = 5, Control *n* = 4, age = 68.9 (not different from previous cohort) and compared supernatant cytokine concentrations with data obtained in our initial experiments (Figure 2). An endotoxin concentration of 1.6EU/mL produced similar levels of cytokine to α-synuclein (monomers or fibrils) for most cytokines thus suggesting a possible confounding effect of endotoxin. However, for IL-1β and IL-18, monomeric α-synuclein had a significantly greater effect than 1.6EU/mL endotoxin (*p* = 0.01), an EU level in excess of the measured level of contaminating endotoxin. A similar pattern was seen for α-synuclein fibrils compared to endotoxin at 1.6EU/mL, but this did not reach statistical significance. Despite the excess production of inflammasome-related cytokines IL-1β and IL-18 by PBMCs stimulated with α-synuclein monomers, there was no corresponding increase in caspase-1 secretion (*p* > 0.05, Figure 2D).

## Discussion

We found that PBMCs collected from both PD patients and age/gender-matched controls stimulated by α-synuclein (both monomeric and fibrillary) produced a robust inflammatory cytokine response. The response was similar in magnitude to LPS stimulation as assessed by both cytokine concentrations in culture supernatant and mRNA expression. Whilst this response may have been confounded by low levels of endotoxin in the α-synuclein preparation, the response of the IL1-β and IL-18 is greater than this low level endotoxin effect, which suggests that α-synuclein may have a specific independent effect on inflammasome-related pathways. Interestingly, it has previously been shown that α-synuclein fibrils (produced from a strain of E. Coli with strongly reduced endotoxicity) stimulate the NLRP3 inflammasome pathway in monocytes to produce IL-1β, in addition to the TLR pathway that is activated by bacterial endotoxin (12). In our study, we found that α-synuclein monomers had a more pronounced effect on IL-1β production than fibrils, but comparison between studies is difficult given the likely variability in aggregate size according to the methodology used to prepare fibrils. We found no significant increase in the PBMC supernatant levels of the inflammasome pathway mediator caspase-1 with α-synuclein stimulation, suggesting that α-synuclein may be acting via caspase-1 independent inflammasome pathways in this setting (26).

In contrast to previous studies (16, 23, 24), there was no evidence of PD-control difference in cytokine production or mRNA expression. Notably, our case-control pairs were well-matched for age and gender and processed in parallel to eliminate variation that may have confounded previous studies. Hence, our data do not support a differential effect of PBMC stimulation in PD cases vs. controls, irrespective of the stimulating antigen. The lack of any patient vs. control differences in cytokine production in response to PBMC stimulation suggests raised levels of inflammatory markers in the serum in PD may relate more to levels of exogenous stimulating antigens or other cytokine sources, rather than to intrinsic properties of the peripheral mononuclear cells. Additionally, oligomeric α-synuclein species might contribute to inflammation in PD but this has not been specifically tested in this study.

The generation of α-synuclein for experimental use typically involves producing recombinant protein in *E.coli*, which invariably leads to endotoxin contamination of the protein product; contamination which can be removed to some extent by cleaning methods, but may remain at low levels and confound cellular processes with sensitivity to endotoxin (27). Our data confirms that even very low level endotoxin levels can have a significant confounding effect. A previous study found that α-synuclein-derived peptides drive specific T-cell responses in PD (11), but it is unclear whether the presence of associated endotoxin had been entirely excluded in these experiments. However, it may be relevant to further study co-stimulation with endotoxin and α-synuclein, given that endotoxin may act synergistically with α-synuclein in TLR stimulation (12), as has been shown in α-synuclein-primed murine microglia (28). Furthermore, endotoxin may influence the conformation of α-synuclein, with different LPS β-sheet content driving alterations in fibril density and changes in associated behavioral phenotypes in animal models (29). However, these mechanisms are not well understood in patients.

A limitation of this study is that the assessment of the PBMC response to varying endotoxin concentrations was undertaken in an independent sub-sample. However, subjects included were similar in age and disease status and the measured cytokine concentrations had minimal between-subject variation suggesting that the responses were representative.

In conclusion, our data suggest that even low levels of endotoxin can confound the measurement of immune cell responses to α-synuclein *in-vitro* and future studies should consider endotoxin quantification. α-synuclein may have independent effects on production of inflammasome-related cytokines, which may perpetuate the immune response in PD. Furthermore, PD and control PBMCs behaved similarly in the face of stimulation in our study which suggests that cell-extrinsic factors may be an important contributor to the chronic inflammation which has been observed in PD. The nature of these agents remains to be fully determined but both α-synuclein and bacterial endotoxins may play a critical role.

## Ethics statement

This study was carried out in accordance with the recommendations of the Health Research Authority East of England-Cambridge Central Research Ethics Committee with written informed consent from all subjects. All subjects gave written informed consent in accordance with the Declaration of Helsinki. The protocol was approved by the East of England-Cambridge Central Research Ethics Committee (REC 03/303).

## Author contributions

AW acquisition of data, analysis and interpretation of data, drafting of the manuscript. RW acquisition of data, critical revision of the manuscript. KS acquisition of data, critical revision of the manuscript. NG preparation of α-synuclein species, acquisition of data, critical revision of the manuscript. HM preparation of α-synuclein species, acquisition of data, critical revision of the manuscript. SH acquisition of data, critical revision of the manuscript. IS acquisition of data, critical revision of the manuscript. RB study supervision, critical revision of the manuscript. CW-G study design and supervision, critical revision of the manuscript.

### Conflict of interest statement

The authors declare that the research was conducted in the absence of any commercial or financial relationships that could be construed as a potential conflict of interest.
