# Supplementary material for: The Peripheral Inflammatory Response to Alpha-Synuclein and Endotoxin in Parkinson's Disease
Source: Front Neurol. 2018 Nov 20;9:946. doi: 10.3389/fneur.2018.00946 (PMC6256248; doi:10.3389/fneur.2018.00946)
Supplement: Supplementary file 1 [file Data_Sheet_1.docx]

| **Variable** | **Patients** | **Paired Controls** | **P** |
| --- | --- | --- | --- |
| **Number (n)** | 31 | 31 |  |
| **Age (years)** | 67.90 ± 6.90 | 67.81 ± 6.14 | 0.954 |
| **Gender**  **(% male)** | 71.0 | 71.9 | 0.610 |
| **Disease duration (years)** | 4.34 ± 1.13 |  | |
| **MDS-UPDRS motor score** | 33.70 ± 2.47 |  |  |
| **Equivalent Levodopa dose** | 620.52 ± 302.40 |  |  |
| **ACE-R score** | 94.10 ± 7.90 |  |  |

*Supplementary Table 1 – Table summarizing basic demographic details of the participants. Values indicate Mean ± SD (Standard deviation); MDS-UPDRS – Movement Disorder Society Unified Parkinson’s Disease Rating Scale; ACE-R – Addenbrooke’s Cognitive Examination (Revised)*
