# Supplementary material for: The Peripheral Inflammatory Response to Alpha-Synuclein and Endotoxin in Parkinson's Disease
Source: Front Neurol. 2018 Nov 20;9:946. doi: 10.3389/fneur.2018.00946 (PMC6256248; doi:10.3389/fneur.2018.00946)
Supplement: Supplementary file 2 [file Data_Sheet_2.docx]

Supplementary methods. Production of α-Synuclein.

α-Synuclein was expressed in Rosetta E. coli (overnight expression at 18°C with IPTG induction), followed by centrifugation in order to pellet the cells. The pellet was then lysed with lysis buffer (20mM NaCl, 50mM Tris pH 7.4, 2mM EGTA, 5mM MgSO4, 5mM DTT and Roche protease inhibitor cocktail) and high-pressure homogenisation (25kPa). Debris was cleared by ultracentrifugation, and after filtering the supernatant was precipitated with 30% ammonium sulphate at 4°C for 30min. Following precipitation, the sample was ultracentrifuged at 39,000g for 30min. The precipitant was dialysed overnight back into an anion exchange buffer. The sample was then loaded on to a HiTrap Q 5ml fast flow column (GE, 17-5053-01). The column was eluted with a 0-1M NaCl continuous gradient on an AKTA purifier (GE Healthcare). Best fractions were pooled and the anion exchange step was repeated. Finally, the eluate was passed through a Superdex 75 size exclusion column (GE healthcare) using an AKTA purifier, into a filtration buffer (HEPES 25mM, NaCl 150mM). Fractions were assessed for protein by coomassie staining on an SDS-PAGE gel, and pooled appropriately before being concentrated and frozen for storage at -80°C. Protein was aggregated by shaking for 5 days at 37°C in a PBS buffer at a concentration of 400μM and sodium azide.
