# Supplementary figures and images for: The Peripheral Inflammatory Response to Alpha-Synuclein and Endotoxin in Parkinson's Disease
Source: Front Neurol. 2018 Nov 20;9:946. doi: 10.3389/fneur.2018.00946 (PMC6256248; doi:10.3389/fneur.2018.00946)

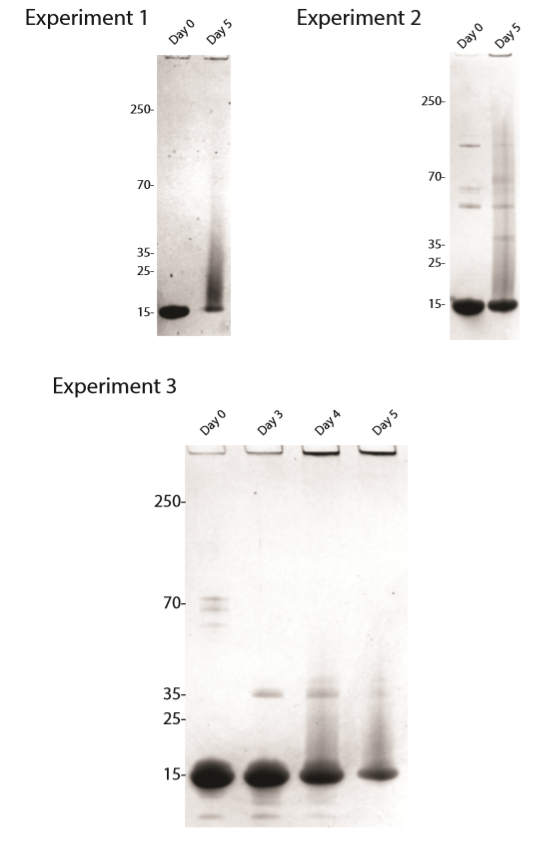


Supplementary Figure 1. SDS-PAGE gel with α-synuclein fractions pre- and post-aggregation.

Supplement: Supplementary file 3 [file Data_Sheet_3.docx]
